# Women's empowerment for active labor: A qualitative study with nurse-midwives in antenatal education for childbirth

**DOI:** 10.18332/ejm/188117

**Published:** 2024-08-22

**Authors:** Marlene I. Lopes, Margarida Vieira, Alexandrina Cardoso

**Affiliations:** 1 Universidade Católica Portuguesa, Centre for Interdisciplinary Research in Health, Faculty of Health Sciences and Nursing, Porto, Portugal; 2 Health Sciences Research Unit, Nursing School of Coimbra, Coimbra, Portugal; 3 Nursing School of Porto, CINTESIS Center for Health Technology and Services Research, Porto, Portugal

**Keywords:** prenatal education, nurse midwives, childbearing women, decision-making, active birth, upright positions

## Abstract

**INTRODUCTION:**

Recognizing the positive impact of movement and positions on labor progression and maternal and neonatal outcomes, there is a strong recommendation to empower women for active labor during antenatal education. This study investigates nurse-midwives' interventions in empowering women for active labor, during antenatal education within primary healthcare settings.

**METHODS:**

A qualitative descriptive study was conducted in Primary Healthcare Units that provide antenatal education for childbirth in Portugal. Semi-structured interviews were conducted with 10 nurse-midwives between August and October 2023. Content analysis, utilizing NVIVO software, was employed for data analysis, and the study adhered to the COREQ reporting guidelines.

**RESULTS:**

Six themes emerged: 1) Perspective of nurse-midwives and contextual influences; 2) Assessment of women's needs; 3) Enhancing women's knowledge; 4) Enhancing women's capabilities; 5) Supporting women in decision-making; and 6) Nurse-midwives' perspective on intervention outcomes. It is necessary to create environments conducive to knowledge and skill acquisition and invest in developing body awareness and its impact on labor progression. Encouraging women's involvement in decision-making is crucial, especially in less flexible hospital environments.

**CONCLUSIONS:**

This study highlighted the value nurse-midwives place on empowering women for active labor. Interventions featured sharing evidence-based practices and birth stories to foster reflection, emphasizing pelvic mobility training and partner involvement. Reflective practices could enable women to explore options and communicate effectively with healthcare professionals during labor.

## INTRODUCTION

Antenatal education is a crucial component of prenatal care, equipping women with the necessary skills to manage labor. It promotes childbirth self-efficacy, and autonomy in decision-making, and enhances confidence in their ability to give birth^1,2^. The freedom for women to assume various positions and move without restrictions during labor influences the positive progression of labor. Mobility and upright positions enhance the gravitational effect on fetal descent by expanding pelvic diameters and increasing the efficacy of uterine contractions, contributing to successful cervical dilation and reducing the first stage of labor by approximately 1 h 20 min. Moreover, it aids in preventing dystocia, lowering the incidence of episiotomy, labor instrumentation, or cesarean section^3^. Evidence also suggests that it has a protective effect on the fetus during labor by optimizing uterine blood flow between the placenta and fetus, decreasing the likelihood of neonatal care unit admission^3^. It is closely tied to a positive birthing experience, linked to a greater sense of involvement in decision-making, autonomy, and self-control during labor. It provides distraction and comfort, potentially reducing the need for analgesia. Additionally, it facilitates the partner’s involvement, contributing to a more positive experience for both^4,5^. It is strongly recommended to promote women’s empowerment, even during pregnancy, by encouraging active participation in decision-making regarding mobility and upright positions^4^. Antenatal education plays a vital role in women’s empowerment, and it is crucial to provide evidence-based information about the risks and benefits of different positions applicable to various contexts. Training opportunities should be made available, preferably involving partners^3,6,7^. The study by Spiby et al.^8^ investigated the reasons women participate in antenatal education and underscored their preference for face-to-face group interactions. It revealed the significant potential for building lasting relationships with peers in similar circumstances, which can help in normalizing their concerns.

In developed countries, there has been a consistent increase in obstetric interventions during labor, underscoring the growing international concern for strengthening women’s empowerment during childbirth^9^. However, making informed decisions and exercising control over their bodies during labor can be challenging, given the influence of paternalistic professional knowledge that often exerts power and control over women, thereby limiting their decision-making capability. Hospital policies imposing restrictions, such as constant surveillance, may infringe upon a woman’s right to choose her preferred positions. Despite receiving information, the confidence to apply this knowledge during labor may not always be present, suggesting that the information provided does not consistently translate effectively into decision-making during labor^6,10^. Therefore, researching to develop and evaluate the effectiveness of interventions promoting women’s empowerment, and enhancing their capacity for choice and control during labor, is crucial for achieving greater satisfaction and improving maternal and neonatal outcomes. It is essential to identify which interventions in antenatal education effectively empower women for active labor. Given the gaps identified in the literature, a study was conducted as part of a larger research project to examine the strategies used by nurse-midwives to empower women for active labor. This study focused on the context of childbirth education within primary healthcare settings.

## METHODS

A qualitative study was conducted to investigate the perceptions and interventions employed by nurse-midwives to empower women for active labor within the context of antenatal education, in primary healthcare settings. Ethical approval was obtained from the Health Ethics Committee of the Centro Regional Health Administration on June 2023 (no. 52/2023). Participants were provided with comprehensive information about the study, informed of their right to refuse or discontinue participation at any point, and provided written informed consent.

Ten community care units (CCUs) in the central region of Portugal, offering a Childbirth Education Program, were selected as the focus of the initial phase of a comprehensive investigation. The overarching goal of this research is to develop and assess an intervention to empower women for active birth in a pilot study. The selection process involved intentional sampling to recruit nurse-midwives from these ten CCUs, with inclusion criteria requiring experience in assisting women in childbirth education for more than 5 years and a track record of implementing interventions that promote active labor. The leader of the research team presented the study during a face-to-face meeting with coordinating nurses from thirteen CCUs and their respective nurse-midwives. Subsequently, the agreement of ten CCUs to participate in the study was obtained. Maternity care in Portugal is primarily provided by physicians and obstetricians. The majority of CCUs have a nurse-midwife and offer free childbirth education to all expectant couples who desire it. All labor units are led by obstetricians and have varying practices related to mobility and upright positions during labor.

Semi-structured individual interviews were carried out between August and October 2023 and were conducted in person by the principal researcher, at a time scheduled based on the preferences of the nurse-midwives, within the CCUs. The selected room provided comfortable conditions, ensuring privacy with only the researcher and the nurse-midwife present. Commencing with a request for sociodemographic information such as age and years of professional experience, the interview followed the structure outlined in the interview guide developed by the research team (Table 1). The interview script underwent a pre-test with two nurse-midwives for refinement. All interviews were recorded in audio and supplemented with field notes and observations of the nurse-midwives’ non-verbal behaviors. Immediate verbatim transcription of the interviews was carried out by the principal researcher after completion. Subsequently, all participants received the transcript of their interview via email for content validation. The study adhered to the proposed criteria for reporting qualitative research (COREQ)^11^.

**Table 1:** Guiding questions for face-to-face semi-structured interviews with ten nurse-midwives at ten community care units

*No.*	*Questions*
1.	What are your views on freedom of movement and positions, especially upright positions, during labor for women with low-risk pregnancies, and how do you perceive the role of antenatal education in facilitating this?
2.	How do you structure your interventions aimed at empowering women for active labor?
3.	In your practice, what specific interventions do you implement, and what strategies do you employ to empower women for active labor?
4.	What facilities or challenges do you face in the implementation of these interventions?
5.	How do you analyze the results of the interventions you use to empower women for active labor?

Data from the interviews were analyzed using the content analysis technique^12^. Transcripts were read and audio listened to several times before coding began, notes were taken and themes and similarities were identified to develop a coding framework. NVIVO software was used to support the analysis process. The themes and categories were refined and finalized after analysis by the research team.

## RESULTS

The interviews had an average duration of 54 minutes, varying between 32 and 79 minutes. The study encompassed ten female nurse-midwives from ten CCUs, aged 39–64 years, with mean age of 47.7 years (SD=8.3). The duration of their nursing careers ranged from 15 to 43 years, averaging 25.1 years (SD=9.2). Additionally, their tenure as nurse-midwives ranged from 8 to 35 years, with an average of 15.1 years (SD=7.6). Their experience in childbirth education ranged from 5 to 32 years, averaging 11.8 years (SD=7.7). Five nurse-midwives also had previous experience in the labor room. The childbirth education sessions were conducted in groups of 6 to 20 women. In five CCUs, the sessions were face-to-face, in three CCUs they were online only, and in two CCUs they were both online and face-to-face. All partners were encouraged to attend.

### Main themes

**Figure 1:**
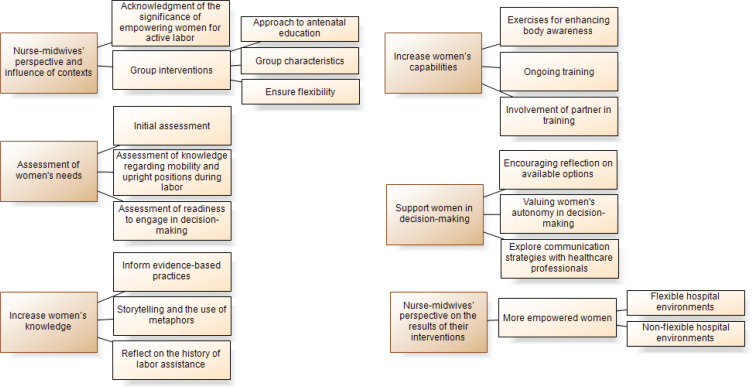
Main themes and subthemes identified from the discourses of ten nurse-midwives across ten community care units

Six main themes emerged from the discourse of the nurse-midwives regarding the empowerment of women to maintain mobility during labor: 1) Nurse-midwives’ perspectives and the influence of contexts; 2) Assessment of women’s needs; 3) Enhancement of women’s knowledge; 4) Enhancement of women’s capabilities; 5) Support for women in decision-making; and 6) Nurse-midwives’ perspectives on the results of their interventions (Figure 1).

### Nurse-midwives’ perspectives and the influence of contexts

All nurse-midwives acknowledge the positive impact of mobility and upright positions on obstetric and neonatal outcomes, as well as on the overall experience of women/couples. They design interventions to empower women for active labor, emphasizing freedom of movement throughout the process. These interventions are implemented in varying ways by nurse-midwives, each recognizing their potential influence on desired outcomes. Seven nurse-midwives structured their groups to maintain consistency with participants in each session, recognizing the benefits, such as fostering closer social bonds. Emphasizing the importance of small group size, eight nurse-midwives considered groups of fewer than 10 women relevant, ensuring greater proximity and individualized attention in their interventions. Regarding the format, five nurse-midwives conducted interventions exclusively in person, two in a hybrid format, and three online. While face-to-face sessions are recognized as the best option, the hybrid format offers increased accessibility for women who are unable to attend in person. The online format is chosen due to constraints on suitable space for face-to-face interventions:

*‘Even though couples also show interest in the online sessions because it provides them with additional flexibility … I have couples who are in the car, coming from work, and are connected. I have couples who have children at home.’* (NM9)

In the online setting, nurse-midwives identified barriers to optimal intervention implementation and evaluation. These included diminished interaction quality between woman and nurse-midwife, as well as constraints in fostering potential social relationships among women, limiting peer learning:

*‘I’ve some women who don’t turn on the camera … I can’t see their expression … I don’t even associate the name with the face.’* (NM7)

*‘There isn’t much sharing, while we, in-person sessions, they sat, relaxed on the balls, sharing stories like, “in my first pregnancy, it was like this, I did it like this”. They shared more, talked more. In the online mode no relationship is established.’* (NM9)

Partners were encouraged to attend, although it is recognized that due to work commitments, they may not always join every session:

*‘It’s quite challenging for partners to attend, on average, only 2 or 3 out of 10 attend all sessions.’* (NM4)

Despite the structured organization, intervention flexibility was maintained through various strategies. This includes executing all planned interventions, or part of them, individually or even implementing them at home, especially in situations of social risk:

*‘We currently have a woman with some cognitive deficit. We thought it best not to include her in this group, we’re planning to provide her with an individual course.’* (NM5)

*‘Sometimes, one or the other expresses a desire to talk to me. In those cases, we extend the session, and I have a oneon-one conversation with that woman in private.’* (NM7)

### Assessment of women’s needs

Nine nurse-midwives consistently conducted individual consultations to assess women’s needs. Six did this in person, one via videoconference, and two during the initial telephone contact with the woman. Regarding this, only one nurse-midwife emphasized the importance of considering women’s expectations and needs for childbirth to plan more personalized interventions. The others primarily prioritized gathering information related to women’s personal and obstetric history. Another nurse-midwife acknowledged that there might be sensitive feelings related to labor that women may not feel comfortable addressing initially. Therefore, ongoing assessment of their needs is essential, along with identifying the most appropriate moments for discussion:

*‘I ask, “On a scale from 0 to 10, what do you feel is your level of confidence for childbirth?”. I follow up by asking “What is needed to reach 10?” to understand what aspects still need to be worked on.’* (NM10)

*‘Sometimes, they don’t share their feelings right away, and it’s in an informal setting that we detect it … I’ve noticed that it’s easier to pick up on certain things like that in informal situations.’* (NM4)

All nurse-midwives noted that most women were unaware of the significance of mobility and upright positions during labor. They often reacted with surprise and disbelief upon learning about the positive impact on labor progression and maternal/neonatal outcomes. This lack of awareness may stem from a general unawareness regarding the importance of their behaviors and attitudes during labor. Understanding the potential of their body in labor and its influence on the birthing process can significantly impact outcomes and satisfaction with the birth experience:

*‘A woman once said to me, “But nurse, are you sure that’s the case? Can I really move?”.’* (NM10)

*‘They’re not fully aware of the power they hold during labor.’* (NM4)

Moreover, a nurse-midwife pointed out the inconsistency that can arise in the statements made by various obstetric health professionals, potentially contributing to women’s lack of information and confidence:

*‘… but when I informed my doctor that I intended to stay mobile during labor, she insisted that delivering the baby while walking was impossible.’* (NM6)

However, it is also acknowledged that an increasing number of women are emerging with enhanced knowledge and a clear desire to actively participate in healthcare decision-making during childbirth. Their examples provide added value for others, offering alternative perspectives and options:

*‘In this group, the woman brought a different perspective, it’s not about being revolutionary, but rather having an alternative viewpoint “No, I don’t think so, I know there’s another possibility, I really want to experience a natural birth, and I don’t want to be confined to a bed, being told what I must do”.’* (NM9)

However, all nurse-midwives noted that this is not the norm. The majority of women tended to rely on healthcare decisions made by professionals in hospital settings due to feelings of insufficient knowledge, undervaluation of their own knowledge compared to professional expertise, and concerns about potentially receiving less attentive care:

*‘I believe most people don’t think that way, there’s this idea that when I go there, they will decide what I’m going to do.’* (NM9)

*‘Some still have that fear … If I express disagreement, what might they think? Will I face reprisals later?’* (NM5)

*‘They are a bit hesitant about creating a birth plan. They fear potential misunderstandings with the hospital team. They want a welcoming environment but are afraid that having a birth plan might be viewed negatively.’* (NM6)

A more paternalistic approach from certain health professionals hinders women’s autonomy in decision-making, even for those who express a desire to exercise it, as highlighted by a nurse-midwife:

*‘I had a woman who created a birth plan and shared it with me. Later, she went to the appointment with the obstetrician and when returned to the course, she was feeling extremely nervous. She mentioned that the obstetrician scolded her and criticized her when she informed him about having made a birth plan with the nurse-midwife.’* (NM6)

### Enhancement of women’s knowledge

Multiple interventions are implemented to enhance women’s awareness of how mobility and upright positions during labor can impact maternal and neonatal outcomes. These interventions include providing information on evidence-based practices and employing diverse strategies. Two nurse-midwives reviewed the history of hospital birth care to help women understand current practices. Four nurse-midwives used films or images to discuss examples of labor that respect physiological processes, and they utilized metaphors to clarify the subjective nature of labor pain. All nurse-midwives engaged in storytelling, where they or women with positive past experiences share their stories. This approach allows for an exploration of different contexts and realities, fostering a comprehensive and reflective understanding that facilitates informed decision-making:

*‘When there’s a lot of confusion, I find an article “Look, I’m going to email this topic that I’m talking about here”, the WHO recommendations. I often send it to them so they understand that this is what’s best for them.’* (NM1)

*‘In the first session, I cover a lot of the history of childbirth so they can understand the current paradigm. I discuss where we came from, from our grandmothers, where we went, to our mothers, and where we are now, the pros and cons of each era … our mothers who had birth experiences without an epidural, in positions that limited blood flow to the abdominal and perineal region, leading to pain. The upright position is completely different, it’s protective.’* (NM10)

*‘I present a film about the world that has several realities, and people end up asking, “Why is that position? Why it’s done like that?”.’* (NM1)

*‘I’m talking about the duration of the contraction and the duration of the pause “Have you seen it? You have all this time to rest.” I use the analogy of the traffic light in labor. What nature brought us was a green and an orange light. In green, we are in the pause; in orange, we are in the uterine wave. But if I’m scared, I’ll increase all those sensations, I’ll be in red, and then I’ll just go back to orange, and then I’m always experiencing pain.’* (NM10)

*‘I also use their testimonies and they tell their stories. The acceptability is different than if I were the one to tell them, and they like it and question it.’* (NM6)

### Enhancement of women’s capabilities

The nurse-midwives consistently emphasized the importance of acquiring skills for a more satisfying labor experience, with a particular focus on freedom of movement. In face-to-face childbirth programs, almost half of the time is dedicated to training in exercises and non-pharmacological strategies for labor pain relief. These interventions often repeat across sessions and may occupy half of a three-hour session or constitute an entire 1.5-hour session devoted exclusively to practicing physical skills for labor. In online childbirth programs, however, this time allocation is typically less. The nurse-midwives advocated for various approaches to positioning and mobilization exercises, such as standing, on the mattress, on a chair, on a birth ball, using a *rebozo*, or engaging in dance. These exercises simulate the labor process, enabling women to recognize sensations and develop experiential knowledge. The active involvement of partners is deemed essential in this training to nurture skills and confidence, both individually and as a couple:

*‘I’ve got the birthing ball, and the women bring a “sarong” for doing the rebozo, suspension, we use the chair, the cushion, the mattress. The partner can be in a chair, or standing, and we incorporate music and dancing. There are multiple strategies.’* (NM1)

*‘Partners need to have this knowledge too because, they’re there advocating for women, offering guidance.’* (NM10)

A nurse-midwife consistently emphasized the importance of promoting women’s awareness of pelvic mobility and its correlation with the baby’s positioning. Encouraging active involvement in labor empowers women with more comfortable and protective movements and positions, fostering the well-being of both the baby and the progression of labor:

*‘They identify the ischia, and I guide them in visualizing it through practical exercises. They place their hands under the rump, feeling for the little bone and visualizing the baby’s head emerging. Then, I provide a model of the pelvis to show precisely where they are touching. Some might say, “No, it doesn’t fit”, and I respond, “Okay if it doesn’t fit, what if we try a leg-opening movement? What happens there? And if we close it, what happens?”.They respond with “Ah, open it!” [with a surprised expression] Alright, I may have a narrow pelvis, but with an active posture and body movement, I can create more space, certain movements can facilitate one type of movement for the baby, and others facilitate another. It’s not a matter of right or wrong, it depends a lot on the labor. In any case, any movement is beneficial.’* (NM10)

All the nurse-midwives emphasized the importance and encourage the training of positions and breathing techniques for childbirth:

*‘I guide them through exercises and encourage reflection. I say, “Notice how much easier this is? We’ll practice birthing in different positions - lying down on the mattress, sitting on the birthing chair, and even squatting”. This helps us understand which positions feel best during labor.’* (NM6)

### Support for women in decision-making

The nurse-midwives implemented interventions to support women in their decision-making about care options related to mobility during labor. They encouraged women to reflect, evaluate diverse care options, examine potential labor scenarios, anticipate risks and benefits, and explore women’s expectations for their childbirth experience within the context of varying hospital environments:

*‘If continuous monitoring is necessary, you can do it while sitting on the birth ball, close to the device, in case there is no Wi-Fi system.’* (NM6)

*‘This and this happens … What actions do you take? And if a particular situation arises, what steps do you take?’* (NM10)

*‘I always strive to present an accurate picture of the destination, so that women don’t form expectations and later express, “I was preparing for a situation that didn’t match what I encountered upon arrival”.’* (NM6)

In this last citation, the nurse-midwife, who had experience in the labor ward, aimed to provide detailed information about the most current practices impacting women’s mobility. This allows women to better prepare for coping with less flexible birth environments.

In this context, two nurse-midwives acknowledged challenges in addressing the significance of mobility during labor. They expressed concern about raising awareness among women regarding the potential disparity between their expectations and the practices in hospital settings. However, it is also acknowledged that women should be informed about all available options, empowering them to make their decisions. Conversely, in hospital environments known for their low flexibility, the emphasis is on maintaining a therapeutic relationship built on trust, preventing conflicts with healthcare professionals, minimizing women’s frustration, and avoiding potential harm to maternal and neonatal outcomes:

*‘You have to handle it very carefully to avoid creating unrealistic expectations for women, right? I can’t just express my opinion or what I think, and then they go into practice and see their expectations disappointed. They should research and clearly understand what they want, seeking institutions that can fulfil their desires or provide the possibilities they are looking for.’* (NM7)

*‘I had to reflect and find a middle ground to keep them calm and prevent additional anxiety, avoiding unrealistic expectations that might not align with the realities they’ll face. Many experienced complications in the postpartum period. What I realized is that situations worsened when they tried to assert their voice. So, I learned to stay calm because the most crucial thing is for them to trust, and I can’t damage that trust in healthcare professionals. Let’s avoid conflicts and work within the feasible conditions.’* (NM8)

Considering instances where a woman’s decision-making may deviate from best practices in labor assistance, such as when she decides to induce labor or have a cesarean section without a medical reason, one nurse-midwife emphasized the importance of understanding women’s choices, respecting their autonomy, and maintaining trust. This is crucial for the progression of labor and ensuring a satisfactory experience:

*‘What’s vital here is our awareness of the choices available and women feeling safe and confident is equally important. What I find crucial is for women to make informed decisions, whatever they may be, and understand that they are responsible for their choices.’* (NM10)

Three nurse-midwives emphasized the significance of exploring strategies with women to enhance assertive communication, aiming to make them feel more at ease and confident when expressing their expectations and concerns to healthcare professionals involved in their labor. The goal is to empower women, granting them greater control and satisfaction throughout the childbirth experience:

*‘You should mention that you feel better when you have the freedom to move, noticing a significant reduction in pain when you go with the rhythm of contractions. This is my approach to empowering women, providing them with tools to feel, understand, and become aware of their bodies. This way, they can communicate with the team in the future, saying, “Hey, it hurts less this way, and I feel better like this, compared to the other way”.’* (NM6)

The importance of respecting women’s autonomy in healthcare decision-making during labor was emphasized by the majority of nurse-midwives. They implement interventions to encourage and support women in making informed decisions, including the promotion of the birth plan as a valuable strategy for reflection and communication with healthcare professionals. One nurse-midwife specifically emphasized the significance of ensuring that the birth plan holds meaningful value for women, cautioning against the use of checklist-type plans that may not align with their individual interests and preferences:

*‘I encourage them to write freely, simply, and clearly, organizing their thoughts by topics. It’s not mandatory, if they feel the need to use a checklist plan and make marks, that’s okay. I just explain that when they mark things, they might be emphasizing aspects that aren’t important to them, while neglecting important things. It’s a way of being influenced by what’s written.’* (NM10)

### Nurse-midwives’ perspectives on the results of their interventions

Regarding the assessment conducted by nurse-midwives on the impact of their interventions, the overwhelming majority asserted that women manifest empowerment through the acquisition of knowledge, skills, and an enhanced sense of childbirth self-efficacy, as reflected in their behaviors during the onset of labor. The importance of this empowerment and involvement in decision-making is crucial and is strongly encouraged, particularly in flexible hospital environments:

*‘I had a woman who shared, “Look, I went to the bathroom to do the exercises, positioned myself on all fours, and held on until the morning”. She then went to the maternity ward, and it all happened quickly. I’ve encountered a few women like that. I recall one who requested a birth ball and ended up teaching others how to use it.’* (NM8)

*‘There was a woman who said, “I arrived, grabbed the side of the bed, and the doctor insisted I lie down. I responded, I’m not lying down because I know I’m fine like this.” [laughs] Nowadays, I sense that women have been more successful because the other side is flexible, but they need to feel empowered, otherwise, it doesn’t always work out.’* (NM10)

Nevertheless, more than a third of nurse-midwives reported the presence of less flexible hospital environments, which restrict women’s freedom of movement and limit their positioning options during labor:

*‘There are still women who tell us that they aren’t allowed to get out of bed because they are being monitored.’* (NM6)

*‘Many of them mention arriving, especially at night or during the onset of labor, and being instructed to lie down.’* (NM8)

## DISCUSSION

This study explored nurse-midwives’ approaches in childbirth education to empower women for active labor. The level of detail in their interventions varied, encompassing the provision of information on the importance of mobility in labor progression and obstetric outcomes, as well as active engagement in exercises fostering reflection and bodily awareness. Many women disclosed a lack of knowledge and reacted with surprise regarding the benefits of mobility and upright positions during labor. Social influences from media, significant others, and interactions with health professionals contribute to women’s perceptions of childbirth, sometimes based on scarce or distorted information. Cultural and historical factors, along with contemporary narratives emphasizing risk and disapproving options outside hospital norms, play a role, in justifying high intervention rates in hospitals, even for women with low-risk pregnancies^13^. Despite these influences, an increasing number of women are seeking knowledge about their birthing options, including the freedom to move during labor. The gap between healthcare professionals’ awareness of women’s information-seeking and their actual practices can create barriers, increasing women’s anxiety and impacting their birth expectations^14^.

Storytelling was considered a strategy to empower women to make informed decisions that reflect their values, and contribute to a deeper understanding of childbirth. By sharing birth stories, women can adjust their expectations, share strategies, explore diverse perspectives on labor, and build support networks. This approach offers more than just information; it facilitates a comprehensive understanding and preparation for childbirth^15,16^. Discussion and dialogue about films showcasing birth experiences outside typical hospital norms were found to be encouraging, facilitating vicarious learning through observation and potential behavior reproduction^17^.

The restorative value of the intervals between contractions for women’s comfort during labor was highlighted. This emphasis on the rest-contraction-rest cycle is key to empowering women and mastering labor management for a positive birth experience. Cutajar et al.^18^ found antenatal education often focuses on contractions’ pain and timing, overlooking the vital rest periods that enable relaxation, confidence, and preparation. Explaining this cycle can enhance women’s labor perceptions and self-efficacy.

Compared to the discourse of nurse-midwives in face-to-face childbirth education programs, those who provide online sessions tend to focus more on delivering information rather than practicing physical skills for labor. Enchelmaier et al.^19^ noted that traditional childbirth education often prioritizes knowledge but also emphasizes the importance of naturally and progressively developing women’s labor skills. This involves repetitive reinforcement of words, positions, and movements to build confidence. Beyond merely acquiring knowledge, these skills emphasize mastering essential techniques for a healthy and satisfying labor experience. The more opportunities women and their partners have to practice physical skills for labor and birth, the more likely they are to utilize them during labor^16,19,20^. Interventions that promote women’s awareness of pelvic mobility, involving the identification of anatomical structures and understanding the impact of certain movements or positions, were highlighted. This is crucial for women’s empowerment, as it allows them to practice and experience various positions and sensations in advance, thereby enhancing their labor preparation. According to Campbell and Nolan^16^, the integration of conscious breathing with rhythmic rocking movements underscores the connection between movement, positioning, and labor progression. This enhances awareness of how movements and postures can modify pelvic diameters and fetal position, and influence decision-making regarding mobility during labor. Nurse-midwives frequently recommend birth ball exercises to improve posture, balance, and labor preparation. The benefits of these exercises are corroborated by randomized studies, which include increased time in vertical positions, a shorter first stage of labor, reduced perception of pain, and enhanced childbirth self-efficacy. This training, which involves practicing on the ball for 20 minutes, three times weekly, over 6–8 weeks also improves body awareness, control, and relaxation, essential for childbirth^21^.

In nurse-midwives’ discourse, there is a notable focus on skills training and mobility awareness for the second stage of labor, while the first stage receives less attention. Interest in women’s mobility during labor is increasing, yet evidence on the most effective childbirth education interventions to empower women for active labor remains scarce. The ‘Labor Hopscotch Framework’, created by midwives, emphasizes the natural process of labor and women’s ability to manage it. It offers a visual guide with practical steps to help women stay active during labor, facilitating optimal fetal positioning essential for a physiological birth^22^.

Despite the interventions designed to support active labor, nurse-midwives acknowledge that hospital settings often limit mobility. This aligns with studies indicating that many hospitals confine women to bed during the first stage of labor. The biomedical model’s dominance restricts movement, favoring routines like fetal monitoring and vaginal exams over mobility^6,23,24^. Women generally perceived these practices as necessary risk reducers, with minimal discussion on their risks or benefits^25^.

Concerning women’s involvement in decision-making, nurse-midwives reported low participation from women, often deferring to professionals’ advice for their and their babies’ well-being. Childbirth expectations, influenced by personal beliefs and prejudices, critically affect women’s decision-making^26^. This complexity underscores the significance of women’s philosophy on childbirth and their confidence in their birthing capabilities. A strong childbirth philosophy and confidence in birthing abilities lead to greater autonomy and less influence from cultural and medical norms^26,27^. Understanding social representations of pregnancy and childbirth is crucial for decision-making, necessitating early birth plan discussions. A meta-synthesis on labor position perceptions highlighted the role of individual and sociocultural factors, including concerns over privacy, negative views on certain positions, and pain expectations. These findings reflect the mixed evidence on position benefits and risks, pointing to a need for more information and support from health professionals, who should offer detailed guidance to enable informed choices, advocating for hospital flexibility to support varied preferences^10^.

This study also highlights efforts by nurse-midwives to inform women about routine healthcare in delivery rooms to reduce anxiety regarding hospital practices, as observed in other studies^17,28^. However, it questions if such intentions might limit women’s awareness of options and best practices, with nurse-midwives facing challenges in providing unbiased information due to hospital constraints. Newnham et al.^29^ found similar issues with epidural information, pointing to the problem of biased informed consent in obstetrics, influenced by culture and hospital norms. This highlights the issue of biased informed consent in obstetric care, where culture and hospital practices shape information. Prenatal education, sometimes rooted in hospital settings, tends to align with institutional needs rather than individual women’s needs. Instead of empowering women to fulfil their expectations, it tends to guide them to align with institutional routines, revealing a growing misalignment between individual and institutional needs within obstetric healthcare, limiting women’s personal choices and experiences^30,31^. Midwives play a key role in offering unbiased information, aiming to empower women for freedom of movement during labor^6^. The nurse-midwives in the current study emphasized the importance of exploring diverse options across hospital contexts for informed decision-making.

The study highlights a gap in customizing care for women desiring a natural birth, suggesting childbirth preparation should cater to each woman’s unique needs and expectations^20^. It emphasizes the importance of shared decision-making, requiring midwives to actively support women in making informed choices. Additionally, it points out the challenges women face in expressing childbirth preferences due to perceived professional dominance, reflecting societal norms that limit decision-making^31-34^. A Spanish study showed that while birth plan counselling, based on shared decision-making, did not increase written birth plans, it led to less epidural use and more diverse pain management strategies, indicating informed decisions^35^. The WHO advocates for women’s right to freedom of movement during labor, urging health professionals to inform them of its benefits and respect their preferences^36^. This emphasizes the role of midwives in promoting physiological labor practices across clinical, educational, political, and social contexts, highlighting the importance of continuity of care from education through labor support to enhance health outcomes. To optimize active birth practices, it is vital to include midwives and consumers in designing birth rooms. This collaborative approach integrates healthcare professionals, expectant mothers, and their families in the design process to ensure the environment supports active birth by meeting professional standards and consumer needs. This method applies user-centered design principles specifically tailored for maternity care, enhancing both safety and comfort.

### Strengths and limitations

This novel study provides a qualitative analysis of nurse-midwives’ perspectives and strategies for empowering women for active labor through antenatal education within Portugal’s primary healthcare system. It includes interviews with ten nurse-midwives across ten community care units, covering intervention content, strategies, challenges, and possibilities. These units, spread across different regions of Portugal, are associated with hospitals noted for varied practices related to women’s mobility during labor, thereby adding diversity to our findings. However, the study has several limitations. The potential bias of nurse-midwives, who might feel evaluated, necessitates caution in interpreting the results. Enriching the research with perspectives from women who received the interventions would have added value, although this was not the main focus. Moreover, challenges in less flexible maternity services that may inhibit desired mobility and upright positions during labor were identified. The study did not address labor ward contexts such as midwife staffing for one-to-one care or continuity of care. Investigating these aspects could clarify the study’s context and deepen our understanding of the issues involved. Extending the study to include nurse-midwives from other contexts might also yield different findings.

## CONCLUSIONS

This study highlighted the consensus on the importance of women’s empowerment in enhancing obstetric and neonatal outcomes, as well as parental satisfaction. The interventions included sharing evidence-based practices, birth stories, and watching films to encourage reflection on diverse experiences. Training in pelvic mobility was emphasized for its benefits to comfort and fetal positioning, with partner involvement also noted as beneficial. Reflective practices enabled women to explore choices and communicate effectively with healthcare providers during labor. These strategies could be adapted to other childbirth education settings to empower women for active labor. However, the findings point to challenges in maternity units that do not support women’s desire for mobility, highlighting the need for further research into childbirth education to enhance women’s empowerment and support their decision-making process in less flexible environments.

## Data Availability

The data supporting this research are available from the authors on reasonable request.
